# Regimes of motion of magnetocapillary swimmers

**DOI:** 10.1140/epje/s10189-021-00065-2

**Published:** 2021-04-24

**Authors:** Alexander Sukhov, Maxime Hubert, Galien Grosjean, Oleg Trosman, Sebastian Ziegler, Ylona Collard, Nicolas Vandewalle, Ana-Sunčana Smith, Jens Harting

**Affiliations:** 1 Helmholtz Institute Erlangen-Nürnberg for Renewable Energy (IEK-11), Forschungszentrum Jülich, Fürther Straße 248, 90429 Nuremberg, Germany; 2 PULS Group, Department of Physics, Interdisciplinary Center for Nanostructured Films, Friedrich-Alexander-Universität Erlangen-Nürnberg, Cauerstraße 3, 91058 Erlangen, Germany; 3 GRASP Lab, CESAM Research Unit, Université de Liège, Allée du 6 Août 19, 4000 Liège, Belgium; 4 IST Austria, Lab Building West, Am Campus 1, 3400 Klosterneuburg, Austria; 5 Group for Computational Life Sciences, Division of Physical Chemistry, Ruđer Bošković Institute, Bijenička cesta 54, P.P. 180, Zagreb, 10002 Croatia; 6 Department of Chemical and Biological Engineering and Department of Physics, Friedrich-Alexander-Universität Erlangen-Nürnberg, Fürther Straße 248, 90429 Nuremberg, Germany

## Abstract

**Abstract:**

The dynamics of a triangular magnetocapillary swimmer is studied using the lattice Boltzmann method. We extend on our previous work, which deals with the self-assembly and a specific type of the swimmer motion characterized by the swimmer’s maximum velocity centred around the particle’s inverse viscous time. Here, we identify additional regimes of motion. First, modifying the ratio of surface tension and magnetic forces allows to study the swimmer propagation in the regime of significantly lower frequencies mainly defined by the strength of the magnetocapillary potential. Second, introducing a constant magnetic contribution in each of the particles in addition to their magnetic moment induced by external fields leads to another regime characterized by strong in-plane swimmer reorientations that resemble experimental observations.

**Graphic Abstract:**

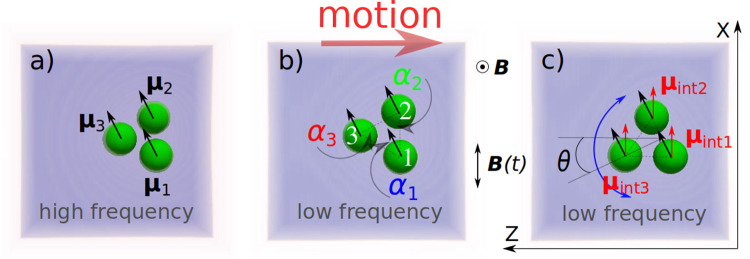

**Supplementary Information:**

The online version contains supplementary material available at 10.1140/epje/s10189-021-00065-2.

## Introduction

Understanding the mechanisms of swimming motion of microorganisms and cells at low Reynolds number is the key to new technologies in biological and medical applications [1–3]. Simultaneously with the study of motion of biological objects like bacteria and sperm cells [4], a new class of microscale devices appeared—artificial or human-made microswimmers. Many of them are designed in a rather simple way, consisting of a number of interacting microscopic particles powered by external excitations, for instance following the framework of the three-beads swimmer [5, 6]. Other examples of artificial microswimmers include magnetically active particles [7], Janus particles [8], particles enduring chemo- [9], visco- [10], gravi- [11] or thermo-taxis [12] or even swarms of microscopic particles mimicking the behaviour of biological organisms [13].

A particular example of an artificial microswimmer capable of self-propelling at a gas/liquid interface is a magnetocapillary microswimmer. Here, several magnetic particles are placed onto an air/water interface. Their assembly is achieved via balancing attractive capillary and repulsive magnetic interactions when a static magnetic field is applied perpendicularly to the fluid interface. The motion is induced by applying periodically altered magnetic fields along the interface, and it can self-propel in a linear [14] or a triangular configuration [15] or perform fully controlled rotations at the interface [16] offering a number of potential applications. These include the transport of cargo particles or interfacial mixing [17].

Although a number of theoretical studies are known for the triangular swimmer configuration [18–20], many of them disregard the presence of the interface or consider external forces only effectively. Here, we study numerically the rich dynamics of magnetocapillary swimmers by taking all relevant effects into account. In Ref. [21], we thoroughly investigated the assembly and the motion of the magnetocapillary swimmer in the regime where the peak velocities of motion are centred at frequencies around the inverse viscous time of a single particle. This regime appears quite different from what is observed in the experiments [15]: (1) we do not observe sizable in-plane rotations of the beads and of the swimmer, (2) the translational amplitudes of the bead motion are significantly smaller, (3) the simulated average velocity of the swimmer is lower than in the experiment. Additionally, the particles detach from the interface and sink at low excitation frequencies, limiting our study to high-frequency regimes.

The current paper aims at a thorough understanding of the parameters that determine the collective motion of the swimmer beads and the propagation efficiency of the full magnetocapillary swimmer. We demonstrate and explain the various modes of motion magnetocapillary swimmers can undergo depending on the precise set-up and choice of parameters. Finally, we demonstrate that our simulations are also able to qualitatively reproduce the strong reorientations of the swimmer as observed in the experiments.

To do so, we reconsider some of the assumptions made in the previous numerical model. For example, in order to prevent sinking, the ratio of the surface tension and the magnetic forces needs to be strongly modified. Furthermore, the assumption of a single magnetic moment is not sufficient to describe the regime observed in the experiments. An additional constant magnetic contribution in each of the particles leads to the experimentally observed in-plane rotations of the particles.

In contrast to simpler and generally computationally less demanding models based, e.g., on analytical solutions of the Stokes equation in combination with a bead-spring dynamics to describe the particle dynamics and their interactions, our approach also allows to investigate the impact of the interface dynamics and the corresponding time-dependent capillary interactions between particles. Additionally, since the lattice Boltzmann method provides a hydrodynamic description at the Navier–Stokes level, inertial effects and the full set of hydrodynamic interactions among particles are naturally taken into account. Furthermore, the treatment of swimmers in geometrical confinement or multiple swimmers is straightforward but beyond the scope of this paper.

The remainder of this article is organized as follows: Sect. 2 deals with the details of the numerical method, and in Sec. 3, different regimes of motion are presented and analysed in depth. Main conclusions on the present and our previous numerical simulations are summarized in the final section.

## Simulation method

The simulation method is thoroughly described in Ref. [21], and we only summarize the main ingredients here. We use a lattice Boltzmann (LB) method for the simulation of fluids [22]. It is based on a discretized version of the Boltzmann equation

1 $$\begin{aligned} \displaystyle f_i^{c}(\varvec{x}+\varvec{c}_i\varDelta t, t+\varDelta t) = f^{c}_i(\varvec{x},t)+\varOmega ^{c}_i(\varvec{x},t). \end{aligned}$$

The latter describes the time evolution of a single-particle distribution function $f^{c}_i(\varvec{x},t)$ at time *t* and position $\varvec{x}$ and $\varvec{c}_i$ denotes the discrete velocity vector in the *i*th direction for fluid component $c=\{1, 2\}$. Here, we use a so-called D3Q19 lattice with $i=1, \ldots , 19$ [23]. The left-hand side of Eq. (1) describes the free streaming of fluid particles, while their collisions are modelled by a Bhatnagar–Gross–Krook (BGK) collision operator on the right-hand side as [24]:

2 $$\begin{aligned} \displaystyle \varOmega ^{c}_i(\varvec{x},t) = - \frac{f^{c}_i(\varvec{x},t)-f^{\mathrm {eq}}_i(\rho ^{c}(\varvec{x},t),\varvec{u}^{c}(\varvec{x},t))}{\tau ^{c}/\varDelta t}. \end{aligned}$$

In Eq. (2), $f^{\mathrm {eq}}_i(\rho ^{c}(\varvec{x},t),\varvec{u}^{c}(\varvec{x},t))$ is a third-order equilibrium distribution function, and macroscopic densities and velocities are given by $\rho ^{c}(\varvec{x},t)=\rho _0\sum _i f^{c}_i(\varvec{x},t)$ as well as $\varvec{u}^c(\varvec{x},t)=\sum _i f^c_i(\varvec{x},t)\varvec{c}_i/\rho ^c(\varvec{x},t)$, respectively ($\rho _0$ is a reference density). $\tau {_{\mathrm {c}}}$ is the relaxation rate of component *c*, which determines the relaxation of $f^{c}_i(\varvec{x},t)$ towards the equilibrium. Space is discretized on a three-dimensional lattice with lattice constant $\varDelta x$, and the time *t* is discretized with $\varDelta t$-steps. The speed of sound $c{_{\mathrm {s}}}=1/\sqrt{3}\varDelta x / \varDelta t$ depends on the choice of the lattice geometry and allows one to obtain the kinematic $\nu ^{c}=c^2_{\mathrm {s}}\varDelta t (\tau ^{c}/\varDelta t - 1/2)$ or the dynamic $\eta ^{c}=\nu ^{c}\rho ^{c}$ fluid viscosities. For simplicity, we set $\varDelta x=\varDelta t=\rho _0=\tau ^{c}=1$ in the remainder of this paper and refer to the units as lattice units (l.u.).

For simulations of the interface and the associated capillary interactions, we choose the pseudopotential method of Shan and Chen and apply a mean-field force between different fluid components as [25, 26]:

3 $$\begin{aligned} \displaystyle \varvec{F}^c_{\mathrm {C}}(\varvec{x},t) = - \psi ^c(\varvec{x},t)\sum _{c'}g_{cc'}\sum _{\varvec{x}'}\psi ^{c'}(\varvec{x}',t)(\varvec{x}'-\varvec{x}).\nonumber \\ \end{aligned}$$

Here, *c* and $c'$ refer to different fluid components, $\varvec{x}'$ denotes the nearest neighbours of the lattice site $\varvec{x}$, and $g_{cc'}$ describes a coupling constant determining the surface tension. $\psi ^c(\varvec{x},t)$ has the form $\psi ^c(\varvec{x},t)\equiv \psi ^c(\rho ^c(\varvec{x},t))=1-\mathrm {e}^{-\rho ^c(\varvec{x},t)}$. The force (3) is applied to the fluid component *c* by adding a shift $\varDelta \varvec{u}^c(\varvec{x},t)=\tau ^c\varvec{F}^c_{\mathrm {C}}(\varvec{x},t)/\rho ^c(\varvec{x},t)$ to the velocity $\varvec{u}^c(\varvec{x},t)$ in the equilibrium distribution. The method is a diffuse interface method, with an interface width of typically 5 lattice sites depending weakly on the coupling strength [27]. In the binary fluid system, we refer to the fluids as “red” (r) and “blue” (b) [28]. In addition, we initialize the system with two equally sized volumes of red and blue fluid, separated by a flat fluid interface.

Three rigid magnetic particles are simulated by solving Newton’s equations of motion for translational and rotational degrees of freedom by means of a leap-frog algorithm. The particles are discretized on the lattice. They are coupled to both fluid species by means of a modified bounce-back boundary condition for both fluid components [28–31].

**Fig. 1:**
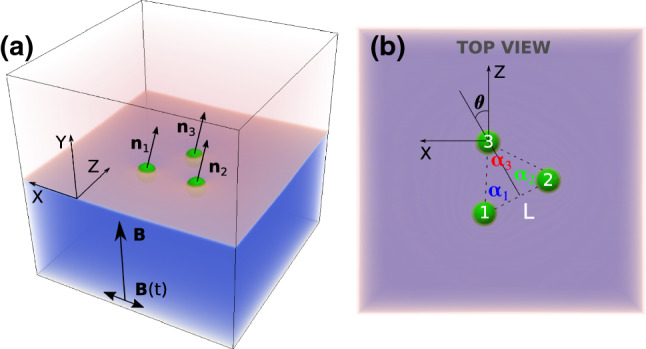
**a** The simulated system including the directions of external magnetic fields ($\varvec{B}$ and $\varvec{B}(t)$) and particle orientation vectors $\varvec{n}_i$, coinciding with the directions of magnetic moments of beads $\varvec{\mu }_i$. **b** Top view of the fluid interface showing angles $\alpha _i$ of the isosceles triangle formed by particles and the orientation of the triangle within the interface $\theta$

A static magnetic field $B_{\mathrm {y}}$ is applied along the positive *y*-direction (see Fig. 1) perpendicular to the interface and induces repulsive magnetic dipolar forces. The repulsion is balanced by an attractive capillary force which is due to the interface deformation caused by the gravity-induced immersion of the particles. This combination of forces allows the assembly of stable particle arrangements at the interface. In analogy with the experiments on magnetocapillary swimmers [15], we choose the amplitude of the time-dependent magnetic field to be approximately three times lower than that of the static field to treat it as a modulation. The field $\varvec{B}(t)=B_{0 \mathrm {x}}\cos \omega t \varvec{e}_{\mathrm {x}}$ causes a deformation of the particle arrangement, which due to collective hydrodynamic interactions leads to the motion of the swimmer under a force free protocol. To properly describe magnetic properties of the particles, a homogeneous external magnetic field $\varvec{B}$ acts on each particle *i* with the magnetic moment $\varvec{\mu }_i$, whose orientation coincides with the material orientation of the particle. The resulting magnetic dipole–dipole interaction between a pair of particles is

4 $$\begin{aligned} \displaystyle U_{ij} = -\frac{\mu _0}{4\pi r_{ij}^3}\left[ 3(\varvec{\mu }_i\cdot \varvec{e}_{ij})(\varvec{\mu }_j\cdot \varvec{e}_{ij})-(\varvec{\mu }_i\cdot \varvec{\mu }_j)\right] . \end{aligned}$$

In Eq. (4), $r_{ij} \equiv \left| \left| \varvec{r}_{ij}\right| \right| \equiv \left| \left| \varvec{r}_i - \varvec{r}_j\right| \right|$ is the distance between the centres of two spheres *i*, *j* located at $\varvec{r}_i$ and $\varvec{r}_j$, respectively, and $\varvec{e}_{ij}$ = $(\varvec{r}_{i}-\varvec{r}_{j})/\left| \left| \varvec{r}_{i}-\varvec{r}_{j}\right| \right|$. The effective magnetic field generated by the magnetic moment $\varvec{\mu }_j$ at the location of another particle *i* is

5 $$\begin{aligned} \displaystyle \varvec{B}_i= - \frac{\partial U_{ij}}{\partial \varvec{\mu }_i} = \frac{\mu _0}{4\pi r^3_{ij}}\left[ 3\varvec{e}_{ji}(\varvec{\mu }_j\cdot \varvec{e}_{ji})-\varvec{\mu }_j\right] . \end{aligned}$$

The resulting magnetic force acting on the *i*th particle is then $\varvec{F}_i= - \varvec{\nabla } \left( - \varvec{\mu }_i\cdot (\varvec{B}_i+\varvec{B})\right)$, or more explicitly

6 $$\begin{aligned} \displaystyle&\varvec{F}_{i} = \frac{3\mu _0}{4\pi r^4_{ij}} \left( \varvec{\mu }_i \left( \varvec{\mu }_j \cdot \varvec{e}_{ji} \right) + \varvec{\mu }_j \left( \varvec{\mu }_i \cdot \varvec{e}_{ji} \right) \right. \nonumber \\&\quad \left. - 5 \varvec{e}_{ji} \left( \varvec{\mu }_j \cdot \varvec{e}_{ji} \right) \left( \varvec{\mu }_i \cdot \varvec{e}_{ji} \right) +\varvec{e}_{ji}(\varvec{\mu }_i \cdot \varvec{\mu }_j)\right) . \end{aligned}$$

We note that the external magnetic field $\varvec{B}$ is homogeneous ($\varvec{\nabla }(\varvec{\mu }_i\cdot \varvec{B})=0)$); hence, the magnetic forces (Eq. (6)) appear solely as a result of the magnetic dipolar interaction. Analogously, the magnetic torque acting on the particle *i* is $\varvec{T}_i=\left[ \varvec{\mu }_i \times (\varvec{B}_i+\varvec{B})\right]$, or explicitly

7 $$\begin{aligned}&\varvec{T}_{i}\!\! = \!\!\frac{\mu _0}{4\pi r_{ij}^3}\cdot \left( 3\left( \varvec{\mu }_j \cdot \varvec{e}_{ji}\right) \left[ \varvec{\mu }_j\!\times \! \varvec{e}_{ji} \right] \right. \nonumber \\&\quad \left. - \left[ \varvec{\mu }_i \!\times \! \varvec{\mu }_j\right] \right) + \left[ \varvec{\mu }_i\! \times \! \varvec{B}\right] .\!\!\! \end{aligned}$$

In the case of three particles, the total force and the total torque for each particle include a summation of expressions (6) and (7) over index *j*. The method with implemented magnetic interactions has already been benchmarked and successfully applied for simulations of magnetocapillary phenomena [32] and swimmers [21].

The following numerical parameters are used throughout the paper: the simulation box consists of $128^3$ cubic cells containing two equally sized fluid lamellae. Rigid walls with midgrid bounce back boundary conditions are placed parallel to the fluid interface, while in any other directions periodic boundary conditions are assumed. All beads have equal radius $R=5\varDelta x$ and density $\rho {_{\mathrm {p}}}=2\rho _0$. The coupling constant $g_{cc'}=0.1$ between the two fluids with densities $\rho _r=\rho _b=0.7\rho _0$ implies a numerical surface tension $\gamma =0.04$ in lattice units. The magnetic moment is chosen in the range $\mu =[1;3]\times 10^5$ in lattice units.

## Results

The equilibrium properties of one, two and three particles at the fluid–fluid interface are thoroughly studied in Ref. [21]. Therefore, here we start directly with three rigid magnetic particles placed at the interface. The particles are in their equilibrium position at a fixed ratio of gravitational and surface tension forces, termed as Bond number, *i.e. Bo*=0.16 (Fig. 1a).

The assembly of the three particles is driven by a time-dependent magnetic field and the main observable of interest is the average velocity of the swimmer, defined as:

8 $$\begin{aligned} \displaystyle \left\langle \varvec{v} \right\rangle =\frac{1}{3}\sum _i \frac{\left( \varvec{r}_i(t_{\mathrm {e}})-\varvec{r}_i(t_{\mathrm {b}})\right) }{(t_{\mathrm {e}}-t_{\mathrm {b}})}, \end{aligned}$$

where $t_{\mathrm {b}}$, $t_{\mathrm {e}}$ stand for the beginning and end times of the external magnetic field action, *i* numbers the particles, and $\varvec{r}_i$ denotes the corresponding coordinates.

In general, the velocities and times can be expressed in relative units related to characteristic processes of the particles at the interface. Since each spherical particle in a fluid experiences a drag force upon translation, its characteristic time to reach the equilibrium can be measured via the coasting or viscous time, defined as $\tau _{\mathrm {cs}}=m/(6\pi \eta R)$ or $\tau _{\mathrm {cs}}=2\rho _{\mathrm {p}}R^2/(9\eta )\approx 95$ $\varDelta t$, where $\rho _{\mathrm {p}}$ is the particle density, *R* is its radius and $\eta$ is the total fluid viscosity. Following the total magnetic field, the particles partly rotate in the fluid, requiring another relevant time scale associated with their rotation. This rotational time can be defined as a ratio of the moment of inertia and the mechanical torque, *i.e.* $\tau _{\mathrm {rt}}=2/5mR^2/(8\pi \eta R^3)$ or $\tau _{\mathrm {rt}}=\rho _{\mathrm {p}}R^2/(15\eta )\approx 29$ $\varDelta t$. One can estimate the time describing the relaxation of the interface as $\tau _{\mathrm {in}}=\eta R/\gamma \approx 44$ $\varDelta t$, where $\gamma$ describes the surface tension. Finally, since the particles are in the magnetocapillary potential, the time related to its strength or effective spring *k* can be approximated using $\tau _{\mathrm {sp}}=2\pi \sqrt{m/k}\approx 65,000$ $\varDelta t$. It is mostly convenient to use one of the shortest time scales associated with the particle for the normalization and the time having fewest variable parameters. Therefore, we express time in units of the coasting time of a single particle $\tau {_{\mathrm {cs}}}$ and the swimmer velocities in diameters per coasting time $(2R)/\tau _{\mathrm {cs}}$.

Following the definition of the coasting time $\tau {_{\mathrm {cs}}}$, we denote *high frequencies* to be in the range of $\omega /(2\pi ) \approx 1/\tau {_{\mathrm {cs}}}$, while the range of *low frequencies* corresponds to $\omega /(2\pi ) \ll 1/\tau {_{\mathrm {cs}}}$.

### Motion at high frequencies

In Ref. [21], we report on the static and some dynamic properties of the magnetocapillary swimmer. It is shown there that the swimmer demonstrates a stable controlled motion for a broad range of swimmer sizes at frequencies in the vicinity of the inverse coasting time $\tau _{\mathrm {cs}}$.

**Fig. 2:**
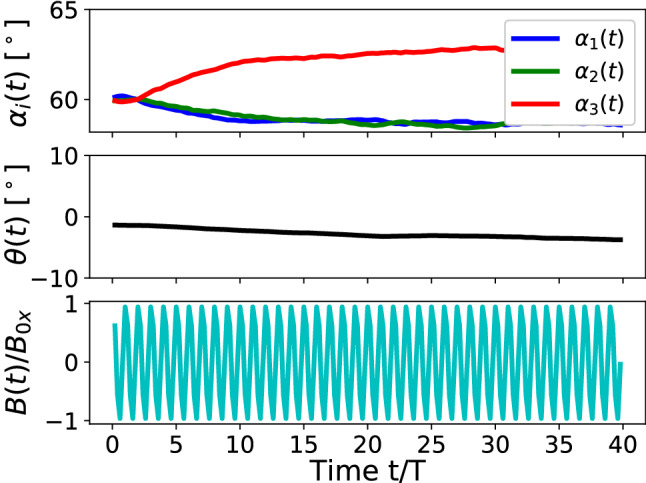
High-frequency time propagation of the inner angles $\alpha _i$ within the triangular swimmer and the orientation angle $\theta$ of the swimmer as defined in Fig. 1b. The lower panel depicts the corresponding reduced time-dependent magnetic field $B(t)/B{_{\mathrm {0 x}}}$. LB-parameters: *Bo* = 0.16, $L=2.3\times (2R)$, $B{_{\mathrm {0 x}}}/B=0.36$, $T=125$ $\varDelta t$

We introduce the angles $\alpha _i(t)$ between the corresponding arms of the swimmer as shown in Fig. 1b as well as the orientation of the swimmer in the plane of the interface $\theta$ defined as the angle between the perpendicular to the line connecting particles 1 and 2 through particle 3 and the *z*-axis (Fig. 1b), where $\theta (t=0)\approx 0$. Starting with an equilateral triangle ($\alpha _i(t_{\mathrm {b}})=60^\circ$, Fig. 2, upper panel), it transforms into an isosceles one for $B(t)\ne 0$, while the triangle as a whole only slightly ($< 10^\circ$) rotates after a number of field periods (Fig. 2, middle panel). Wishing to demonstrate the short period of the external magnetic field (Fig. 2, lower panel), we note that on a longer time scale the rotation illustrated by the angle $\theta (t)$ is not transient and remains of the order of several degrees.

**Fig. 3:**
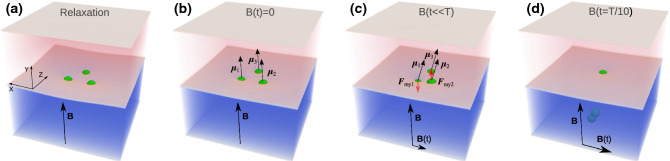
Demonstration of the irreversible sinking of particles during the swimmer motion. **a** shows the swimmer during its relaxation (*B*(*t*) is not applied), **b** illustrates the state directly after the driving *B*(*t*)-field is switched on, **c** shows the swimmer just before the sinking of the base particles and **d** demonstrates the state of the degraded swimmer, *i.e.* when two particles are detached from the interface. Parameters of simulations: the Bond number *Bo* = 0.16, the ratio of magnetic fields $B{_{\mathrm {0x}}}/B\approx 0.57$, the period of the external field is $T=10^5$ $\varDelta t$

Approaching lower frequencies in this regime often leads to a sinking of one or two particles, thus destroying the swimmer. This effect is very pronounced at moderate and large swimmer sizes ($L> 3 \times 2R$), hindering the study of its motion at low frequencies. In experiments on magnetocapillary swimmers (Ref. [15]), sinking of particles was never observed, raising the question of proper parameters in the LB-simulations. When magnetic forces are applied upon the swimmer motion, the Bo-number carefully chosen in our previous study [21] is not the only parameter defining the particles’ vertical position. Indeed, one can consider the ratio of surface tension and magnetic dipolar forces $F_{\mathrm {st}}/F_{\mathrm {mg}}=2\pi \gamma R/(\mu _0\mu ^2/(4\pi r^4_{\mathrm {pp}}))$, where $\mu _0$ is the magnetic permeability of vacuum, $\mu$ is the total bead magnetic moment, and $r_{\mathrm {pp}}$ is the distance between the particle centres. Aiming at the maximum of the magnetic force, thus taking $r_{\mathrm {pp}}=2R$, we find the ratio in the experimental situation to be $F_{\mathrm {st}}/F_{\mathrm {mg}}\Big |_{\mathrm {ex}}\approx 10^4$ and in LB-simulations of the order of $F_{\mathrm {st}}/F_{\mathrm {mg}}\Big |_{\mathrm {LB}}\approx 1$. It is obvious from this estimate that in the experiments the surface tension dominates over magnetic interactions, while in the simulations, the forces are of the same order. In addition to its strength, the magnetic force is strongly dependent on the mutual orientation of interacting magnetic moments (Eq. (6)). In particular, if the particles are in one plane and their magnetic moments are aligned strictly perpendicular to the interface (Fig. 3a, b), the out-of-plane magnetic force is zero. If, however, the magnetic moments become tilted by the external time-dependent magnetic field (Fig. 3c), the out-of-plane components of the magnetic forces beat the surface tension forces detaching the particles from the interface (Fig. 3d). Interestingly, only the particles along the $\varvec{B}(t)$-field vector (particles 1 and 2) sink, since by symmetry particle 3 does not experience any out-of-plane magnetic force in this configuration.

### Motion at low frequencies

In order to get closer to the experimental regime and to avoid the sinking of particles, the ratio of the forces $F_{\mathrm {st}}/F_{\mathrm {mg}}$ needs to be increased. A natural way to increase this ratio is by decreasing the magnetic moment. This does not work, however, since it reduces the magnetic repulsion and leads solely to the aggregation of particles. Alternatively, the surface tension could be increased, but the computational effort required to increase the surface tension by several orders of magnitude is prohibitive. We did perform simulations with a twice as high resolution of the particle corresponding to a similar scaling of the surface tension, but did not find any qualitative difference in the swimmer movement. Further solutions like reducing the strength of the time-dependent field amplitude would significantly reduce the swimmer velocity and increase the computational effort enormously.

We therefore numerically set the out-of-plane component of magnetic forces to zero, and thus effectively increase the ratio of surface tension to magnetic force. This solution is physically sound since all magnetic dipolar forces are pair forces and the total force remains zero.

**Fig. 4:**
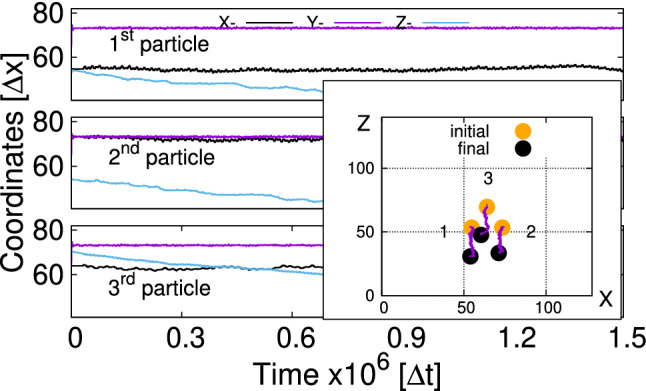
Trajectories of each bead during the swimmer motion in the regime of low frequencies. The inset shows the initial and final positions of the swimmer on the interface. LB-parameters: *Bo* = 0.16, $L=1.8\times (2R)$, $B{_{\mathrm {0x}}}/B=0.36$, T = 20,000 $\varDelta t$

As shown in Fig. 4, the swimmer in this regime propagates in the direction perpendicular to the oscillation of the magnetic field. This aspect is similar to the previously observed motion [21]. The way it propagates is, however, different. The amplitudes of particle oscillations are significantly larger than before and reach values around $0.3\times 2R$. Also, all three beads experience pronounced oscillations and not only particles 1 and 2 as it is the case at high frequencies [21].

**Fig. 5:**
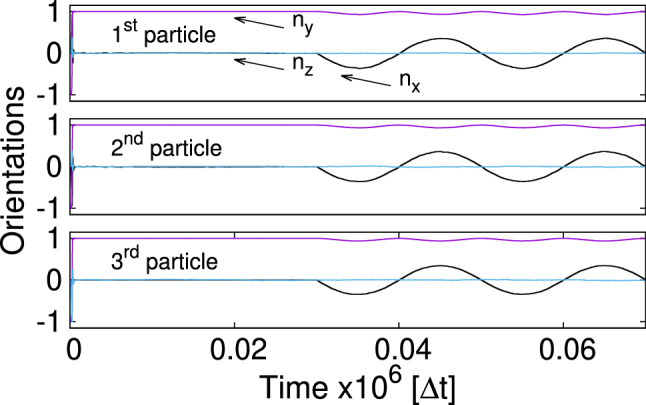
Trajectories of orientation vectors $\varvec{n}_i$ (Fig. 1a) for each bead in the regime of low frequencies. LB-parameters: *Bo* = 0.16, $L=1.8\times (2R)$, $B{_{\mathrm {0x}}}/B=0.36$, $T=20,000$ $\varDelta t$. The *B*(*t*)-field is applied after $t_{\mathrm {b}}=$30,000 $\varDelta t$

Figure 5 shows the trajectories of orientation unit vectors $\varvec{n}_i$ stressing the fact that orientations of the particles follow the direction of the time-dependent external magnetic field which is applied after a relaxation time of $t_{\mathrm {b}}=30,000$ $\varDelta t$. As mentioned in Ref. [21], the time $t_{\mathrm {b}}$ is chosen after studying vertical relaxations of single and multiple particles at the fluid–fluid interface. It assures that for $t > t_{\mathrm {b}}$ the vertical motion of both the particles and the fluid is negligibly small. The maximum declination of the direction vector $n_{\mathrm {x} i}^{\mathrm {max}}\approx 0.36$ is the consequence of the applied time-dependent and static magnetic fields $B{_{\mathrm {0 x}}}/B\approx 0.36$. Since direction vectors are unit vectors $|\varvec{n}_i|=1$ and the magnetic fields are applied in the xy-plane, the $n_{\mathrm {z} i}$-component remains nearly zero attaining the maximum declination for $\varDelta n_{\mathrm {y} i}=1-\sqrt{1-n_{\mathrm {x} i}^2}\approx 0.07$ (see Fig. 5 for $n_{\mathrm {y} i}$).

**Fig. 6:**
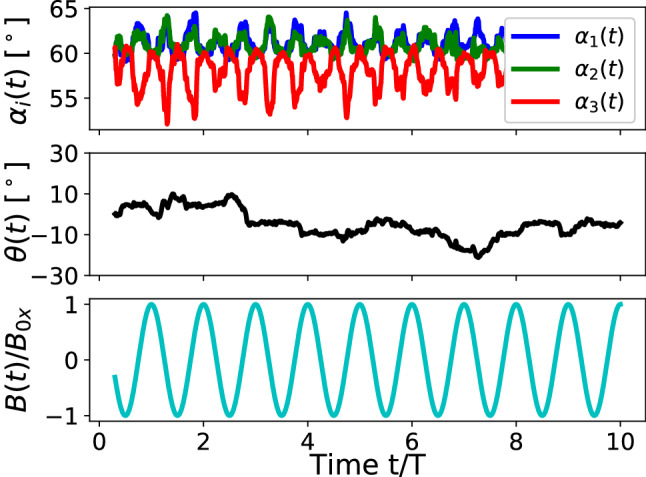
Time propagation of the inner angles $\alpha _i$ within the triangular swimmer and the orientation angle $\theta$ of the swimmer as defined in Fig. 1b. Low panel demonstrates the time propagation of the time-dependent magnetic field $B(t)/B{_{\mathrm {0 x}}}$. LB-parameters: *Bo* = 0.16, $L=1.5\times (2R)$, $B{_{\mathrm {0x}}}/B=0.36$, T = 100,000 $\varDelta t$

The orientation of the swimmer $\theta$ in this case does not show any regular pattern (Fig. 6), but it indicates an overall slight rotation of the triangle with respect to the initial orientation by less than $10^\circ$. Since during the motion of the swimmer $\theta$ reaches values exceeding $20^\circ$, we conclude that the swimmer in this regime tries to synchronize its orientation with respect to the driving *B*(*t*)-field. Figure 6 demonstrates significant differences in the time dependence of angles $\alpha _i$ compared to the high-frequency mode. Here, angle $\alpha _3$ associated with the third particle periodically decreases, while angles $\alpha _1$ and $\alpha _2$ increase to the same amount. This is the consequence of the reduced magnetic moments $\varvec{\mu }_1$ and $\varvec{\mu }_2$ on the average of the field period, hence a reduced magnetic repulsion between the particles. As a result, the attractive capillary interaction pushes particles 1 and 2 closer to each other compared to the situation in equilibrium ($\alpha _i=60^\circ$).

The fact that the frequencies of angle oscillations are two times higher than the ones related to the driving magnetic field reflects the magnetic pair-interaction nature. Indeed, the induced magnetic moment can be written as $\varvec{\mu }=\chi V_0/\mu _0 (\varvec{B}+\varvec{B}(t))$ or simply $\mu \sim \mathrm {const}+\cos \omega t$, where $\chi$ is the magnetic susceptibility and $V_0$ is the volume of a particle. If the magnetic moments are oriented nearly perpendicular to the interface, then the magnetic repulsion force scales as $F_{12}\sim (\varvec{\mu }\cdot \varvec{\mu })=(\mathrm {const} + C_1 \cos \omega t + C_2 \cos 2\omega t)$, where $C_{1, 2}$ are some physical constants. In other words, the second harmonics are inherent in magnetic interactions and since each particle interacts with two others at the same time, several first and second harmonics are always present in their trajectories with different weights.

**Fig. 7:**
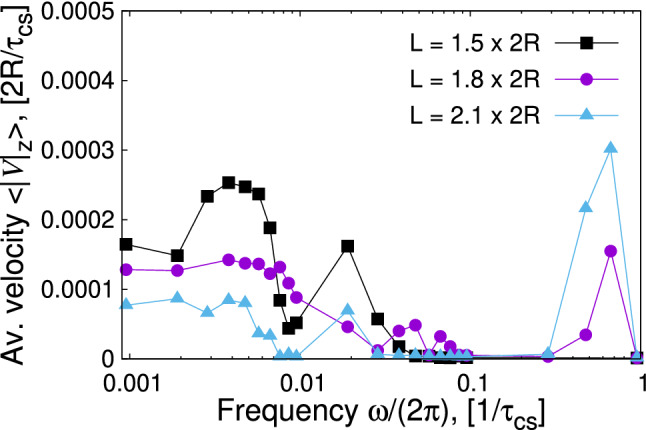
Velocity of the centre of mass of the swimmer averaged over multiple periods versus frequency of the external magnetic field in the regime of low frequencies. The swimmer propagates mainly along the *z*-axis, a drift along the *x*-axis can be ignored. LB-parameters: *Bo* = 0.16, $B{_{\mathrm {0x}}}/B=0.36$

Figure 7 summarizes the behaviour of the average velocity of the swimmer as a function of the frequency of the external field *B*(*t*) for different swimmer sizes *L*. In general, swimmer velocities are sensitive variables. Therefore, the swimming velocity results from averaging in time and on different numerical trajectories, namely: first, the swimmer velocity is calculated according to the definition given by Eq. (8) which describes a time average of the displacement of the swimmer’s centre of mass. Second, for every swimmer size *L* we average over two trajectories differing in the time when the *B*(*t*) is applied, *i.e.* $t_{\mathrm {b}}=30000$ $\varDelta t$ and $t_{\mathrm {b}}=100000$ $\varDelta t$, meaning that initial positions of the swimmer in both cases are slightly different. This averaging allows to reduce the effect of the slightly changing particle discretization on the velocity measurement. Corresponding error bars would be smaller than the symbols and are thus omitted.

Compared to the similar dependence of the regime at high frequencies (Fig. 9a in Ref. [21]), we clearly identify the swimmer operation in a much broader range of frequencies for all swimmer sizes.

Figure 7 also represents the swimmer motion at low and moderate frequencies of its driving. It captures the whole complexity of the motion in terms of capillary and magnetic interactions (magnetocapillary potential), hydrodynamic interactions, the behaviour of the interface, triangular geometry of the swimmer and the effects associated with the inertia of the particles. Although there is a number of studies dealing with the physics of swimmer motion [5, 6, 18, 20, 33–35], it is hardly possible to include all the aforementioned effects in a single theoretical formalism. Studies relying on the force-based approach suggest that the maximum swimmer velocity should be centred around the frequencies associated with the harmonic potential controlling the arm length, *e.g.* $\omega _{\mathrm {St}}=k/(6\pi \eta R)$ [18, 20].

The low-frequency behaviour originates from the resonance due to the particle mass and the magnetocapillary potential, such that we can estimate the associated frequencies using the expression for a harmonic oscillator $\omega _{\mathrm {spring}} \sim \sqrt{k/m}$. Appendix A provides a simplified way of extracting the spring constants $k_{12 x}$ from the particle trajectories of the swimmer of moderate sizes. The maximum velocities associated with the magnetocapillary potential quantified by effective spring constants *k* fall into the range $\omega (\langle V\rangle ^{\mathrm {max}}) \approx [0.0005 ; 0.009]$ $1/\tau _{\mathrm {cs}}$. Figure 7 confirms these estimates by showing broad velocity distributions at different swimmer sizes between [0.001; 0.01] $1/\tau _{\mathrm {cs}}$. A decay of averaged velocities as a function of the swimmer size *L* in this $\omega$-range reflects decreasing hydrodynamic interactions upon the swimmer growing, which is shown by expression (43) of Ref. [20] though in the Oseen-tensor representation only ($R/L<1/6$). If extended to the sizes considered here ($1/4<R/L<1/3$, Rotne–Prager tensor), it confirms the observed behaviour of $\langle V^{\mathrm {max}}(L)\rangle$ for $\omega \in [0.001; 0.01]$ $1/\tau _{\mathrm {cs}}$.

At frequencies around the inversed coasting time, the pronounced peaks are related to the relaxation of single beads in the fluid. This feature was observed and studied in Ref. [21]. The origin of further peaks at moderate frequencies is speculative. We expect them to stem from higher harmonics induced by magnetic interactions.

### Motion at low frequencies and finite internal magnetic moment

The simulated triangular magnetocapillary swimmer presented so far shows how its motion differs depending on the applied field frequency $\omega$, properties of the interface or the swimmer size *L*. Compared to the experimental situation in which high in-plane cyclic bead rotations are observed (Fig. 6 in Ref. [15]), the simulated swimmer never shows such type of motion since the propagation of $\theta$ in Fig. 6 is not periodic. Indeed, the strong in-plane bead rotations observed in the experiments point to more complex magnetic properties of the beads.

For unraveling the magnetic properties of the beads, a series of experiments was performed using a single particle placed at the interface and driven by an external magnetic field [16]. Therein, the magnetic bead rotates under the application of a constant magnetic field in the plane of the interface when the field rapidly changes its orientation by $180^\circ$. Assuming that the magnetic moment is of paramagnetic nature, *i.e.* $\varvec{\mu } \sim \varvec{B}$, the associated magnetic torque on the particle should be zero ($\varvec{T}\sim [\varvec{\mu } \times \varvec{B}]=0$) and cannot cause the particle to rotate around its own axes. This fact leads to the hypothesis of the existence of a permanent internal magnetic moment that is randomly oriented when the particle is placed at the interface. Upon switching the field orientation from “$+$” to “−”, the permanent internal magnetic moment follows the field and mechanically rotates the particle. Moreover, a correct linear scaling was experimentally observed for the maximum rotation frequency of the bead with respect to the magnetic field amplitude (Fig. 8 in Ref. [16]).

The origin of the small constant internal magnetic contribution in the particles is still under debate [16]. Taking into account their size (diameters of several hundred micrometers) and almost perfect spherical form, it can be shown by exact numerical micromagnetic simulations (Sec. 5 in Ref. [16]) that their net magnetic moment should be zero in the absence of an external field. The latter should also be true in much larger systems, *i.e.* above diameters 1–3 $\mu$m for which the micromagnetic simulations were performed. At large particle sizes ($> 1\mu$m) long-ranged magnetic dipolar interactions start favouring the formation of magnetic domains that are randomly oriented in space and their number grows upon reaching hundreds of micrometers. Considering that the particles are highly monodisperse in density [16], only two effects can cause the presence of a finite internal magnetic moment: (i) defects at the boundaries of some magnetic domains (similar to the *Barkhausen effect*) and/or (ii) the fabrication process of the magnetic beads. In the latter case, steel wires are originally cut into small cylinders, then pressed into spherical dies and finally rounded [16]. We speculate that this process might induce additional magnetic anisotropies in the particles.

**Fig. 8:**
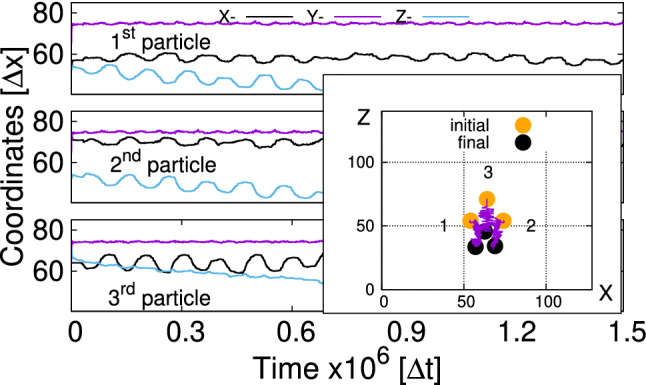
Trajectories of each bead during the swimmer motion in the regime of low frequencies and finite internal magnetic moment. The inset shows the initial and final positions of the swimmer on the interface. LB-parameters: *Bo* = 0.16, $L=1.5\times (2R)$, $B{_{\mathrm {0x}}}/B=0.36$, T = 100,000 $\varDelta t$, $\mu {_{\mathrm {int x}}}=0.1\mu$, $\mu {_{\mathrm {int y}}}=0$, $\mu {_{\mathrm {int z}}}=0$

**Fig. 9:**
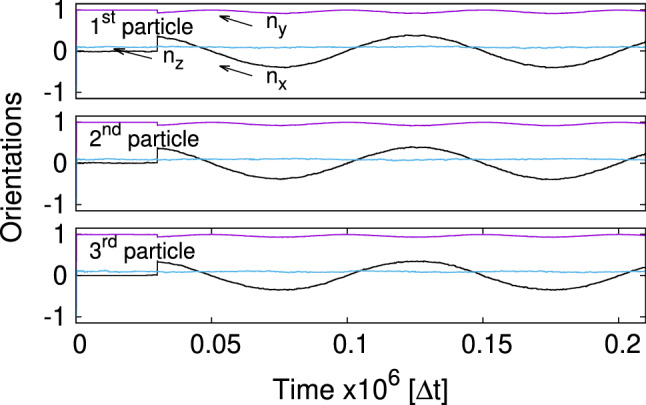
Trajectories of orientation vectors $\varvec{n}_i$ (Fig. 1a) for each bead in the regime of low frequencies and finite internal magnetic moment. LB-parameters: *Bo* = 0.16, $L=1.5\times (2R)$, $B{_{\mathrm {0x}}}/B=0.36$, T = 100,000 $\varDelta t$, $\mu {_{\mathrm {int x}}}=0.1\mu$, $\mu {_{\mathrm {int y}}}=0$, $\mu {_{\mathrm {int z}}}=0$. The *B*(*t*)-field is applied after $t_{\mathrm {b}}=$30,000 $\varDelta t$

Being equipped with the experimental proof for the existence of the permanent internal magnetic moment, we assume that the total magnetic moment in each particle has two magnetic contributions

9 $$\begin{aligned} \displaystyle {\varvec{\mu }{_{\mathrm {tot}}}_i = \varvec{\mu }_i + \varvec{\mu }{_{\mathrm {int}}}_i}, \end{aligned}$$

where $\varvec{\mu }_i$ is the main magnetic contribution, while $\varvec{\mu }{_{\mathrm {int}}}_i$ is a smaller magnetic contribution which is not parallel to the external magnetic field $\varvec{B}$.

Appendix B provides full details of how magnetic forces and torques are modified if Eq. (9) holds. In particular, magnetic forces gain three additional terms, since internal magnetic moments interact with the induced ones and with themselves in different particles. The same applies to the magnetic torques. For the strength of the internal magnetic moment, we rely on experimental observations [16], where the strength was estimated to be approximately in the range $\mu {_{\mathrm {int}}}\in [0.1; 0.15]\mu ^{\mathrm {max}}$ of the maximum induced magnetic moment.

Figure 8 represents the motion of the swimmer at low frequencies and in the presence of finite internal magnetic moment $\mu {_{\mathrm {int x}}}=0.1 \mu$ ($\mu {_{\mathrm {int y}}}=0$, $\mu {_{\mathrm {int z}}}=0$) in each bead. Each of the three particles has the same internal magnetic moment in strength and direction and the induced magnetic moment is present as described above. There is a notable difference in the way how all particles move in this case with respect to previous modes (Fig. 4): both x- and z-components of the particles along which the *B*(*t*)-magnetic field is applied experience sizable oscillations, while the top particle performs oscillations only along the x-direction. The net swimmer displacement in this regime is approximately the same as in the absence of the internal magnetic moment (inset of Fig. 8).

The dynamics of rotation vectors $\varvec{n}_i$ ($\mu {_{\mathrm {int x}}}=0.1 \mu$, Fig. 9) does not essentially differ from that when the internal magnetic moment is zero (Fig. 5). We only witness a very tiny z-component of $\varvec{n}_i$ for all particles which is attributed to a new in-plane magnetic equilibrium due to the presence of $\mu {_{\mathrm {int x}}}$.

**Fig. 10:**
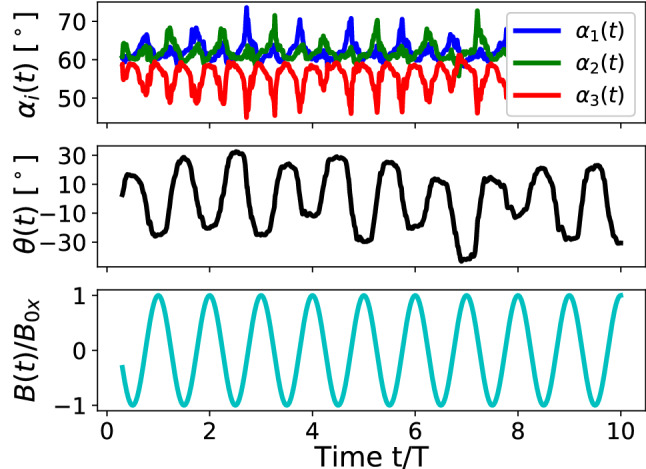
Time propagation of the inner angles $\alpha _i$ within the triangular swimmer and the orientation angle $\theta$ of the swimmer as defined in Fig. 1b. Low panel demonstrates the time propagation of the time-dependent magnetic field $B(t)/B{_{\mathrm {0 x}}}$. LB-parameters: *Bo* = 0.16, $L=1.5\times (2R)$, $B{_{\mathrm {0x}}}/B=0.36$, T = 100,000 $\varDelta t$, $\mu {_{\mathrm {int x}}}=0.1\mu$, $\mu {_{\mathrm {int y}}}=0$, $\mu {_{\mathrm {int z}}}=0$

The presence of the finite internal magnetic moment leads to substantial in-plane dynamics of all the beads within the swimmer. As shown in Fig. 10, the orientation of the swimmer $\theta$ follows exactly the period of the external magnetic field *B*(*t*) and on the large time scale the swimmer keeps its in-plane orientation such that $\int _0^{t\rightarrow \infty }\theta (t)dt\approx 0$. Additionally, we observe that the strength of $\mu {_{\mathrm {int}}}$ defines how strong the angle $\theta$ deviates from the equilibrium $\theta =0$ meaning that one can judge about the magnitude of $\mu {_{\mathrm {int}}}$ based on $\langle \theta ^{\mathrm {max}}\rangle$. Along with the pronounced $\theta (t)$-dependence, we detect several changes in the propagation of $\alpha _i(t)$. In contrast to the triangle deformations in the absence of $\mu {_{\mathrm {int}}}$ (Fig. 6), where all angles $\varDelta \alpha _i^{\mathrm {max}}<5^\circ$, we now notice larger triangle deformations $\varDelta \alpha _i^{\mathrm {max}}\approx 10^\circ$ that again depend on the magnitude of $\mu {_{\mathrm {int}}}$. Moreover, an asymmetric propagation of the base angles $\alpha _1$ and $\alpha _2$ (Fig. 10, upper panel) complies with the asymmetry introduced by the direction of the internal magnetic moment $\mu {_{\mathrm {int x}}}$: the x-components of the induced and the internal magnetic moments are aligned anti or parallel depending on the *B*(*t*)-direction.

Noteworthy is also the orientation of the internal magnetic moment. The dynamics presented in Fig. 10 is valid for the x- or in general an in-plane component of $\mu {_{\mathrm {int}}}$. Once one introduces $\mu {_{\mathrm {int y}}}$ or an out-of-plane component solely, which is additive to the induced magnetic moment, we do not observe periodic reorientations of $\theta$ (not shown here).

Although quantitatively there might be differences between our simulations and the experimental observations for angles $\alpha _i$ and $\theta$ (*e.g.* Fig. 6 in Ref. [15]), qualitatively we recover the main experimental findings: when introducing a smaller in-plane component of the internal magnetic moment $\mu {_{\mathrm {int}}}\approx 0.1\mu ^{\mathrm {max}}$, the swimmer demonstrates remarkable reorientations defined by the angle $\theta$ which follow the external *B*(*t*)-field by its simultaneous swift propulsion at the interface.

**Fig. 11:**
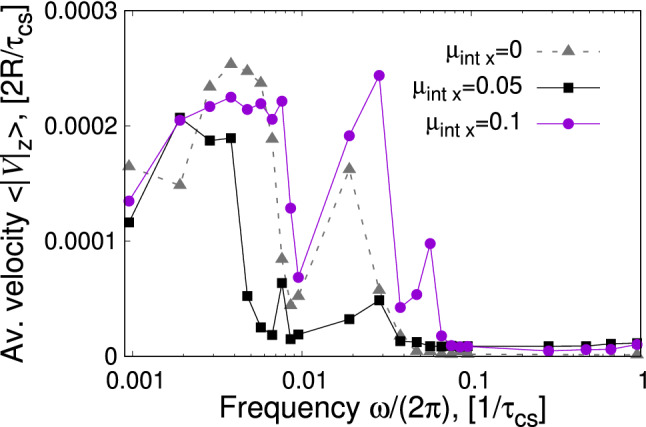
Velocity of the centre of mass of the swimmer averaged over multiple periods versus frequency of the external magnetic field in the regime of low frequencies and finite internal magnetic moment. LB-parameters: *Bo* = 0.16, $L=1.5\times (2R)$, $B{_{\mathrm {0x}}}/B=0.36$

Finally, the dependence of the averaged velocity on the applied frequency (Fig.  11) does not change significantly with respect to the situation with the absent internal magnetic moment (Fig. 7), *i.e.* the swimmer is most efficient for frequencies $\omega /(2\pi )\sim [0.001; 0.01]$ $1/\tau _{_{\mathrm {cs}}}$. The average velocity does not acquire any boost or decrease compared to the situation shown in Fig. 7. The only difference concerns the range of high frequencies around the inverse coasting time, where no swimming is observed in the presence of the $\mu {_{\mathrm {int x}}}$ moment (Fig. 11).

## Summary and discussion

### Different regimes of motion

Using the lattice Boltzmann method with the Shan–Chen model for the fluid–fluid interface, we demonstrate three different regimes of stable swimmer motion: the regime with magnetic particles at high (i) and low (ii) frequencies and (iii) the regime of magnetic particles with a small internal ferromagnetic contribution at low frequencies.

In regime (i) (Ref. [21]) the magnetic moments of all particles follow a set of externally applied static and oscillating magnetic fields. The swimmer propagates having small particle displacements and shows neither typical sizable in-plane rotations of the beads as observed in the experiments [15] nor periodic reorientations of the swimmer (evolution of $\theta$ in Fig. 2). The peaks of the averaged swimmer velocity (Fig. 11 in Ref. [21]) are observed at high frequencies characteristic to viscous or coasting times of the particles. Reduction of the driving frequency required for a better temporal resolution of the motion often led to sinking of one or several particles (Fig. 2), thus destroying the swimmer. We find that sinking is caused by sizable out-of-plane components of magnetic forces exceeding the surface tension force.

Regime (ii) is achieved through suppressing the vertical component of the magnetic force of the swimmer having otherwise the parameters of regime (i). As a result, each bead attains amplitudes up to its radius and the swimmer is capable to propagate at significantly lower frequencies associated with the strength of the magnetocapillary potential. A good temporal resolution of its motion is accomplished. The swimmer in this regime shows sizable side deformations; however, no periodic reorientations are characterized by the angle $\theta$ (Fig. 6).

Finally, regime (iii) is primarily characterized through the existence of an additional small constant internal magnetic contribution that is evidenced in the experiments [16]. The swimmer demonstrates a motion at low characteristic frequencies and possesses typical $\theta$-reorientations (Fig. 10) similar to those observed experimentally [15]. It should be noted that only the in-plane component of the internal magnetic moment causes the typical swimmer motion seen experimentally, while the out-of-plane magnetic contributions do not lead to any sizable swimmer reorientations, since in this case it adds to the vertical magnetic moment.

In regimes (ii) and (iii), we witness one remarkable non-trivial effect associated with the frequency of the driving field. Despite the external magnetic field $\varvec{B}(t)$ having only one frequency $\omega$, the associated magnetic force might have a second harmonic, since the magnetic force between each pair of particles is calculated according to $F_{ij}\sim (\varvec{\mu }_i\cdot \varvec{\mu }_j)\sim \mathrm {const}+\cos \omega t +\cos 2\omega t$ for each magnetic moment driven by the total field $\varvec{B}+\varvec{B}(t)$. This frequency doubling effect is clearly seen in Figs. 6 and 10, when comparing the inner angle- (upper panels) and the driving field (low panels) propagations. This feature should be kept in mind in case of magnetocapillary or, in general, any magnetically driven swimmer, when applying theoretical *e.g.* bead-spring models: the force will contain $2\omega$ although the field is applied with a single frequency $\omega$.

### Swimmer velocities

In experiments, for bead diameters $2R=500$ $\mu$m the average swimmer velocity reaches values up to $\langle V{_{\mathrm {exp}}}\rangle \approx 0.3$ (2*R*)/*T* [15] for the ratio of oscillating to static field $B{_{\mathrm {0 x}}}/B\approx 0.5$ and about $\langle V{_{\mathrm {exp}}}\rangle \approx 0.02$ (2*R*)/*T* for moderate $B{_{\mathrm {0 x}}}/B\approx 0.1$. Our LB-simulations yield for the maximum average velocities $\langle V{_{\mathrm {LB}}}\rangle$ $\approx 0.0004$ (2*R*)/*T* in regime (i) [21] and approximately $\langle V{_{\mathrm {LB}}}\rangle \approx 0.06$ (2*R*)/*T* in both regimes (ii) and (iii) (Sects. 3.2 and 3.3, respectively). Although the simulated velocities in absolute units are of the same order of magnitude in all the described regimes ($\langle V{_{\mathrm {LB}}}\rangle \approx 10^{-5}$ l.u.), we reach a better agreement with the experiment in units of (2*R*)/*T* for regimes (ii) and (iii). It is also in line with the analytical predictions for the triangular swimmer velocity in Ref. [20] (Eq. (43)) or for a dumbbell swimmer including effects of inertia (Ref. [35], Eq. (2)): the lower the potential constant is (estimates in Appendix A yield $k\approx 10^{-5}$ l.u.), the lower are the frequencies of the peak velocities leading to a better time resolution and the higher are the maximum velocity amplitudes. Finally, we note that since the swimmer velocity typically scales quadratically $\langle V\rangle \sim A^2$ [18, 20, 35, 36] with the external driving amplitude *A*, this is also the way to tune up the velocity. Note that *A* is the magnetic force amplitude and not the magnetic AC-field amplitude, for which our LB-simulations yield a biquadratic velocity-vs-field dependence. With triangular magnetocapillary swimmers, it has, however, a limitation at increasing field ratios, since at values $B{_{\mathrm {0 x}}}/B\approx 0.4$ a dynamic transition from a triangular to a linear swimmer configuration occurs (Fig. 2 of Ref. [17]). We are able to reproduce this transition in our LB-simulations and therefore fix the ratio around $B{_{\mathrm {0 x}}}/B\approx 0.36$ to assure the triangular form [21].

### Simulation method and parameters

The presented simulations of magnetocapillary swimmers are a challenging task. On the one hand, we model the fluid–fluid interface and its dynamics coupled with the dynamics of the externally driven magnetic particles. On the other hand, the magnetic properties of the swimmer are included in the simulation by taking into account not simply effective external repulsive forces but the different magnetic contributions to the total magnetic moment of the beads leading to the particle repulsion. As a consequence, such thorough modelling of the problem allows for very detailed insights into the static properties of magnetocapillary swimmers such as horizontal and vertical positioning of the beads upon swimmer self-assembling, the conditions for which the particles may detach from the interface and a realistic description of capillary phenomena for finite particle sizes and moderate inter-particle distances [21]. Moreover, the rich physics of the swimmer propagation associated with the potential strength and the interface dynamics is reflected in their velocity vs frequency dependencies (Figs. 7,  11).

At the same time, the choice of the method and the limitation in reaching realistic surface tensions with acceptable computational effort are also responsible for the sinking of particles upon the swimmer motion (Fig. 3). It helps better understand the experimental conditions such as a very high surface tension that practically pins the floating particles to the interface permitting thus only in-plane particle dynamics. A possible solution of the problem associated with the sinking of beads in LB-simulations consists in modifying not the interface, but rather in setting the vertical components of magnetic forces to zero, thus suppressing the out-of-plane swimmer dynamics.

Furthermore, a number of parameters have a strong impact on the propagation of the magnetocapillary swimmer. First, the particle radius *R* which should be larger than the thickness of the diffuse interface ($\approx 5\varDelta x$)  [28, 37] and large enough to provide a spatial resolution required to reproduce the correct surrounding flow field. At the same time, *R* has to be small enough to assure a comparison with the experimental situation, where the radius is small compared to the system size. Particularly, we found that increasing the particle radius to values larger than $5\varDelta x$ has only a minor influence on the swimmer velocity. Second, in view of simulations of long-ranged capillary phenomena, the total size of the simulated fluid or the size of the simulation box is very crucial. Using periodic boundary conditions in lateral directions of the box (Fig. 1a), the box side length should be large to ensure saturation of the interface from the point of contact with the particles towards the edges. Although the LB-method scales nicely to large processor counts, very large system sizes require enormous computational times. Examination of the box size on the swimmer velocity showed less than a factor two. The third parameter that should be carefully chosen is the *Bo*-number, which can be tuned either by the particle density or by its radius. For a better interface resolution, a notably curved interface profile is desired; hence, large *Bo*-numbers, while exceeding $Bo^{\mathrm {crit}}\approx 0.21$, lead to sinking of particles. Taking into account the listed criteria and the available computational resources is the base for our choice of parameters as given at the end of Sec. 2.

## Conclusions and outlook

We demonstrated that our LB simulations are capable of reproducing the rich dynamics of magnetocapillary microswimmers by taking into account all relevant physical ingredients. We proved in particular that the existence of small ferromagnetic contributions in the particle properties ($\varvec{\mu }{_{\mathrm {int}}}\ne 0$) captures the characteristic swimmer reorientations observed experimentally [15]. Moreover, we claim that when the magnetization of the beads is only induced by an external magnetic field ($\varvec{\mu }\ne 0$, $\varvec{\mu }{_{\mathrm {int}}}=0$), the swimmer is also capable of swimming and its motion is then characterized by the maximum swimmer velocity to be centred around the particle’s inverse coasting time in the range of higher driving frequencies. For lower driving frequencies and a high ratio of surface tension to magnetic forces, the swimmer motion is determined by the strength of the magnetocapillary particle interactions.

As an outlook, yet another regime of motion might be numerically studied. In that case, an additional small static magnetic field is applied along the *z*-axis (Fig. 1) leading to sizable *individual* rotations of each bead in the plane of the interface. In this setup, a swift swimmer motion is reached experimentally presumably because of strong hydrodynamic flows [17]. Furthermore, we plan to investigate in detail the effect of the particle and fluid inertia on the swimmer motion.

### Supplementary Information

Below is the link to the electronic supplementary material.

Supplementary material 1 (avi 6885 KB)

## Figures and Tables

**Fig. 12:**
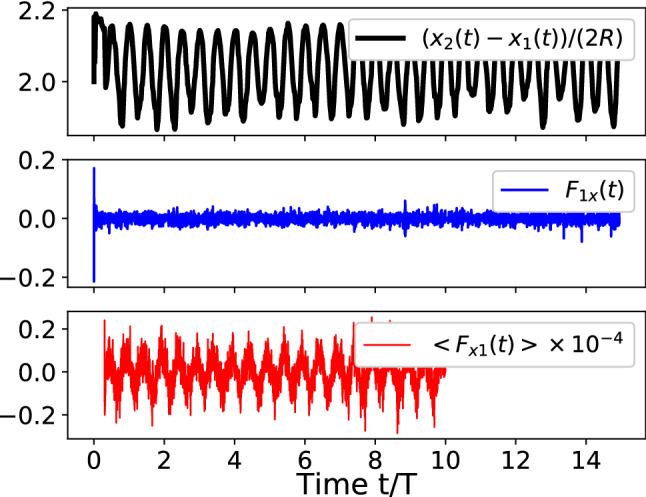
Extraction of spring constants characteristic for the motion of particles 1 and 2 within the swimmer. Upper panel: mutual displacement $(x_2(t)-x_1(t))/(2R)$ of two particles normalized by the bead diameter. Middle panel: non-averaged force $F_{x 1}(t)$. Low panel: Averaged force acting on particle 1. LB-parameters: *Bo* = 0.16, $L=2.1\times (2R)$, $B{_{\mathrm {0x}}}/B=0.36$, T = 100,000 $\varDelta t$

## Appendix A: Calculation of spring constants

For the spring potential between *e.g.* particles 1 and 2 (Fig. 1) defined as

A.1 $$\begin{aligned} \displaystyle \phi (\varvec{r}_1-\varvec{r}_2)=\phi _{12}=\frac{1}{2}k \left( |\varvec{r}_1-\varvec{r}_2|-L\right) ^2, \end{aligned}$$

with *L* being the equilibrium distance between the centres of particles, we define the force acting on particle 1 via

A.2 $$\begin{aligned} \displaystyle \varvec{F}_1(t)= - \varvec{\nabla }_1 \phi (\varvec{r}_1-\varvec{r}_2). \end{aligned}$$

In general, spring constants have different components $k_x$, $k_y$ and $k_z$, so that

A.3 $$\begin{aligned} \begin{aligned} \displaystyle \varvec{F}_1(t)= -&\left( 1-\frac{L}{|\varvec{r}_1(t)-\varvec{r}_2(t)|}\right) \times \\&\begin{pmatrix} k_{1x} &{} 0 &{} 0 \\ 0 &{} k_{1y} &{} 0 \\ 0 &{} 0 &{} k_{1z} \end{pmatrix} \begin{pmatrix} x_1(t)-x_2(t) \\ y_1(t)-y_2(t) \\ z_1(t)-z_2(t) \end{pmatrix}. \end{aligned} \end{aligned}$$

The x-component is defined according to Eq. (A.2) as

A.4 $$\begin{aligned} \displaystyle k_{1x} = \frac{-F_{1x}(t)}{\left( 1-\frac{L}{|\varvec{r}_1(t)-\varvec{r}_2(t)|}\right) \left( x_1(t)-x_2(t)\right) }. \end{aligned}$$

The time average is defined using

A.5 $$\begin{aligned} \displaystyle \langle k_{1x}\rangle = \frac{1}{t_{\infty }-t_0}\int _{t_0}^{t_{\infty }}\frac{-F_{1x}(t)dt}{\left( 1-\frac{L}{|\varvec{r}_1(t)-\varvec{r}_2(t)|}\right) \left( x_1(t)-x_2(t)\right) }.\nonumber \\ \end{aligned}$$

Using expression (A.5), one can determine *e.g.* $\langle k_{12 x}\rangle$ between particles 1 and 2 from their trajectories upon the swimmer motion. The unit for the *k*-constant in LB-simulations is $[\rho _0 \frac{\varDelta x^3}{3\varDelta t^2}]$.

Figure 12 demonstrates how the effective spring constant $k_{12 x}$ related to the interaction between particles 1 and 2 can be calculated. For this the relative displacement between particles 1 and 2, $x_2(t)-x_1(t)$ is steadily measured, while the swimmer moves (Fig. 12, upper panel). At the same time, the total force acting on particle 1 is recorded (Fig. 12, middle panel) and averaged over the period of the external magnetic field $2\pi /\omega$. Inserting the obtained expressions for the force and the mutual displacements into Eq. (A.5), we obtain the values of $k_{12 x}(L)$ as a function of the swimmer size. Since the capillary potential gets very distorted at low swimmer sizes, the expressions of total averaged forces $\langle F_{x 1}(t)\rangle$ are very noisy. For moderate and large swimmer sizes ($L\approx 2\times (2R)$) the picture is represented by Fig. 10 and yields values of the order $k_{12 x}\approx 10^{-5}$ l.u.

## Appendix B: Implementation of magnetic forces in case of a finite internal magnetic moment

In the experiments on magnetocapillary swimmers [15], there are indications of the existence of an additional permanent magnetic moment, such that the total magnetic moment of each particle reads

B.1 $$\begin{aligned} \displaystyle \varvec{\mu }{_{\mathrm {tot}}} = {\varvec{\mu } + {\varvec{\mu }}{_{\mathrm {int}}}}, \end{aligned}$$

whereby the magnetic moment $\varvec{\mu }$ is larger than the internal magnetic moment $\mu {_{\mathrm {int}}}\ll \mu$.

Thus, the force exerted by the moment $\varvec{\mu }{_{\mathrm {tot}}}_j$ on the magnetic moment $\varvec{\mu }{_{\mathrm {tot}}}_i$ is

B.2 $$\begin{aligned} \displaystyle \varvec{F}_{ji}^{\mathrm {magn. tot}} = -\varvec{\nabla } \left( -({\varvec{\mu _i}}+{\varvec{\mu }{_{\mathrm {int}}}_i}) \cdot \varvec{B}{_{\mathrm {tot}}}_j\right) , \end{aligned}$$

where

B.3 $$\begin{aligned} \begin{aligned} \displaystyle \varvec{B}{_{\mathrm {tot}}}_j/\left( \frac{\mu _0}{4\pi |\varvec{r}|^3_{ij}}\right) =&\left[ 3\varvec{e}_{ij}({\varvec{\mu }_j}\cdot \varvec{e}_{ij})-{\varvec{\mu }_j}\right] \\&+\left[ 3\varvec{e}_{ij}({\varvec{\mu }{_{\mathrm {int}}}_j}\cdot \varvec{e}_{ij})-{\varvec{\mu }{_{\mathrm {int}}}_j}\right] . \end{aligned} \end{aligned}$$

The resulting force exerted by the moment $\varvec{\mu }{_{\mathrm {tot}}}_j$ on the magnetic moment $\varvec{\mu }{_{\mathrm {tot}}}_i$ is

B.4 $$\begin{aligned} \begin{aligned}&\displaystyle \varvec{F}_{ji}^{\mathrm {magn. tot}}/ \left( \frac{3\mu _0}{4\pi |\varvec{r}_{ji}|^4}\right) = \\&\quad \left( {\varvec{\mu }_i} \left( { \varvec{\mu }_j} \cdot \varvec{e}_{ji} \right) + {\varvec{\mu }_j} \left( {\varvec{\mu }_i} \cdot \varvec{e}_{ji} \right) \right. \\&\quad -\left. 5 \varvec{e}_{ji} \left( {\varvec{\mu }_j} \cdot \varvec{e}_{ji} \right) \left( {\varvec{\mu }_i} \cdot \varvec{e}_{ji} \right) +\varvec{e}_{ji}({\varvec{\mu }_i} \cdot {\varvec{\mu }_j})\right) \\&\quad +\left( {\varvec{\mu }_i} \left( {\varvec{\mu }{_{\mathrm {int}}}_j} \cdot \varvec{e}_{ji} \right) + {\varvec{\mu }{_{\mathrm {int}}}_j} \left( {\varvec{\mu }_i} \cdot \varvec{e}_{ji} \right) \right. \\&\quad - 5 \varvec{e}_{ji} \left( {\varvec{\mu }{_{\mathrm {int}}}_j} \cdot \varvec{e}_{ji} \right) \left( {\varvec{\mu }_i} \cdot \varvec{e}_{ji}\right) \\&\quad \left. +\varvec{e}_{ji}({\varvec{\mu }_i} \cdot {\varvec{\mu }{_{\mathrm {int}}}_j})\right) \\&\quad +\left( {\varvec{\mu }{_{\mathrm {int}}}_i} \left( {\varvec{\mu }_j} \cdot \varvec{e}_{ji} \right) + {\varvec{\mu }_j} \left( {\varvec{\mu }{_{\mathrm {int}}}_i} \cdot \varvec{e}_{ji} \right) \right. \\&\quad \left. - 5 \varvec{e}_{ji} \left( {\varvec{\mu }_j} \cdot \varvec{e}_{ji} \right) \left( {\varvec{\mu }{_{\mathrm {int}}}_i} \cdot \varvec{e}_{ji}\right) +\varvec{e}_{ji}({\varvec{\mu }{_{\mathrm {int}}}_i} \cdot {\varvec{\mu }_j})\right) \\&\quad +\left( {\varvec{\mu }{_{\mathrm {int}}}_i} \left( {\varvec{\mu }{_{\mathrm {int}}}_j} \cdot \varvec{e}_{ji} \right) + {\varvec{\mu }{_{\mathrm {int}}}_j} \left( {\varvec{\mu }{_{\mathrm {int}}}_i} \cdot \varvec{e}_{ji} \right) \right. \\&\quad \left. - 5 \varvec{e}_{ji} \left( {\varvec{\mu }{_{\mathrm {int}}}_j} \cdot \varvec{e}_{ji} \right) \left( {\varvec{\mu }{_{\mathrm {int}}}_i} \cdot \varvec{e}_{ji}\right) +\varvec{e}_{ji}({\varvec{\mu }{_{\mathrm {int}}}_i} \cdot {\varvec{\mu }{_{\mathrm {int}}}_j})\right) . \end{aligned} \end{aligned}$$

Similarly, magnetic torques should read

B.5 $$\begin{aligned} \displaystyle \varvec{T}_{ji}^{\mathrm {magn.tot}} = \left[ \left( {\varvec{\mu }_i}+{\varvec{\mu }{_{\mathrm {int}}}_i}\right) \times \left( \varvec{B}_j+\varvec{B}{_{\mathrm {int}}}_j+\varvec{B}\right) \right] . \end{aligned}$$

The resulting total magnetic torque is

B.6 $$\begin{aligned} \begin{aligned} \displaystyle&\varvec{T}_{ji}^{\mathrm {magn. tot}}/ \left( \frac{\mu _0}{4\pi |\varvec{r}_{ji}|^3}\right) = \\&\left( 3\left( {\varvec{\mu }_j} \cdot \varvec{e}_{ji}\right) \left[ {\varvec{\mu }_i}\times \varvec{e}_{ji} \right] - \left[ {\varvec{\mu }_i} \times {\varvec{\mu }_j}\right] \right) +\\&\left( 3\left( {\varvec{\mu }{_{\mathrm {int}}}_j} \cdot \varvec{e}_{ji}\right) \left[ {\varvec{\mu }_i}\times \varvec{e}_{ji} \right] - \left[ {\varvec{\mu }_i} \times {\varvec{\mu }{_{\mathrm {int}}}_j}\right] \right) +\\&\left( 3\left( {\varvec{\mu }_j} \cdot \varvec{e}_{ji}\right) \left[ {\varvec{\mu }{_{\mathrm {int}}}_i}\times \varvec{e}_{ji} \right] - \left[ {\varvec{\mu }{_{\mathrm {int}}}_i} \times {\varvec{\mu }_j}\right] \right) +\\&\left( 3\left( {\varvec{\mu }{_{\mathrm {int}}}_j} \cdot \varvec{e}_{ji}\right) \left[ {\varvec{\mu }{_{\mathrm {int}}}_i}\times \varvec{e}_{ji} \right] - \left[ {\varvec{\mu }{_{\mathrm {int}}}_i} \times {\varvec{\mu }{_{\mathrm {int}}}_j}\right] \right) +\\&\left[ \left( {\varvec{\mu }_i}+{\varvec{\mu }{_{\mathrm {int}}}_i}\right) \times \varvec{B}\right] /\left( \frac{\mu _0}{4\pi |\varvec{r}_{ji}|^3}\right) . \end{aligned} \end{aligned}$$
